# DETERMINANTS OF SETTLEMENT INTENTIONS AMONG OLDER CHINESE INTERNAL MIGRANTS: AN INTEGRATED PERSPECTIVE

**DOI:** 10.1093/geroni/igae098.0211

**Published:** 2024-12-31

**Authors:** Rui Kang, Jia-jia Zhou

**Affiliations:** The Hong Kong Polytechnic University, Hong Kong; Hong Kong Baptist University, Hong Kong, Hong Kong

## Abstract

Understanding factors driving settlement intention of older migrants is important for policies to promote successful integration. This research examined to what extent the adaption, attachment to hometown, and environmental factors affect the settlement intentions among older internal migrants in China. A cross-sectional survey was conducted among 598 older migrants aged 50 and above in Shenzhen and Guangzhou, two large cities in China. Data on settlement intention, Hukou transfer intention, multidimensional adaption, environmental factors and attachment to hometown were collected using questionnaire survey. Multinomial logistic regression modelling was employed to identify the determinants of settlement intention. The findings revealed that better psychological adaptation and frequent participation in community activities increased the likelihood of settling in the host city and transferring Hukou registration. Regular involvement in voluntary activities and increased access to welfare services decreased the probability of expressing reluctance to settle and transfer their Hukou status. Conversely, maintaining connections with friends from their hometown reduced the likelihood of settlement in host city. Older migrants with living parents in their hometown are more likely to resist settle in the host city. However, a higher perceived quality of physical environments in the neighborhood elevated their likelihood of not deciding to settle and transfer their Hukou status. It is crucial for policy and service interventions to focus on enhancing the welfare and service environments in neighborhoods. Such improvements can facilitate the successful integration of older migrants into their new communities, thereby promoting their well-being and contributing to the overall social cohesion.

